# Longitudinal Linkages between Older and Younger Sibling Depressive Symptoms and Perceived Sibling Relationship Quality

**DOI:** 10.1007/s10964-019-01009-y

**Published:** 2019-03-08

**Authors:** Kirsten L. Buist, Marloes S. van Tergouw, Hans M. Koot, Susan Branje

**Affiliations:** 1 Department of Clinical Child and Family Studies, Utrecht University, P.O. Box 80140, Utrecht, TC 3508 The Netherlands; 2 Department of Clinical, Neuro and Developmental Psychology, Vrije Universiteit Amsterdam, Amsterdam, The Netherlands; 3 Department of Youth and Family, Utrecht University, Utrecht, The Netherlands

**Keywords:** Adolescents, Siblings, Sibling relationship quality, Depressive symptoms

## Abstract

The sibling relationship has an important impact on children’s emotional functioning, but it is yet unclear whether and how sibling relationship quality affects adolescent depressive symptoms over time. This study contributes to existing knowledge by examining the relative importance of three aspects of sibling relationship quality (i.e., support, conflict and power balance) on the one hand and sibling depressive symptoms on the other hand in predicting adolescent depressive symptoms over time. Additionally, this study examined whether these influence patterns were moderated by perceived sibling relationship quality and by dyadic gender composition. Across six annual waves, 412 Dutch adolescents (57% boys; *M*_age_ = 12.34 years) and their older siblings (47% boys; *M*_age_ = 15.36 years) reported on depressive symptoms and sibling relationship quality. Cross-lagged panel analyses showed that only sibling depressive symptoms and not perceived relationship quality predicted adolescent depressive symptoms one year later. This effect was not moderated by sibling relationship quality or gender composition. These results indicate that sibling depressive symptoms may be a risk factor for adolescent depressive symptoms.

## Introduction

The sibling relationship has an important impact on children’s emotional functioning, as it is often one of the most long-lasting relationships throughout the lifespan and siblings spend considerable time together (Noller 2005). The relationship between siblings is generally described as ambivalent due to the presence of positive (e.g., support) as well as negative characteristics (e.g., conflict and power imbalance) (Deater-Deckard et al. 2002). During adolescence, the sibling relationship changes, in that it often becomes more intimate (McHale et al. 2006) and more equal concerning power (Tucker et al. 2010), but also more conflictive (Campione-Barr and Smetana 2010). At the same time depressive symptoms increase during adolescence (Costello et al. 2011), and these may be strongly influenced by family relationships (Cyranowski et al. 2000). Earlier studies have demonstrated that siblings affect each other’s emotional wellbeing (Buist et al. 2011). Additionally, adolescent depressive symptoms may be a precursor for adult major depression (Thapar et al. 2012). Therefore, it is important to examine to what extent the sibling relationship influences depressive symptoms during adolescence.

### Sibling Relationships and Depressive Symptoms

During adolescence, siblings can influence each other’s depressive symptoms in two ways. First, adolescents’ depressive symptoms may be influenced by characteristics of the relationship with their sibling, for example by the quality of the relationship. Attachment theory (Bowlby 1969) suggests that children will show more negative developmental outcomes, such as depressive symptoms, when they have poor interpersonal relationships. Whereas perceived sibling support may have a direct positive effect as well as protect against adverse effects of adolescent changes, a sibling relationship characterized by high levels of conflict and power imbalance and perceived lack of support may result in more depressive symptoms due to resulting negative emotions, loneliness and lowered self-esteem. Empirical research has indeed shown that more sibling support is related to less adolescent depressive symptoms (Branje et al. 2004; Killoren et al. 2017), whereas more conflict between siblings is related to more adolescent depressive symptoms (Moser and Jacob 2002; Vogt Yuan 2009). Although power is considered to be an important aspect of the sibling relationship (Campione-Barr 2017), this aspect has rarely been examined (Buist et al. 2013; Caspi 2011). However, recent (cross-sectional) work has demonstrated that adolescents who report higher levels of power imbalance in the sibling relationship also report significantly higher levels of depressive symptoms (Buist et al. 2017). Given the fact that the sibling relationship is uniquely characterized by support as well as by conflict and power balance, this study will examine these three dimensions of relationship quality in relation to adolescent depressive symptoms.

Second, siblings may also influence each other by a process of sibling identification in which siblings learn behaviors through observation and interaction (Bank and Kahn 1982). One of the reasons siblings may be effective models is that they are similar to each other (Whiteman et al. 2011). Siblings may mimic each other’s emotional states, including depressive behaviors, as they frequently and intensively spend time together. Previous research has provided some empirical support for this pattern and has shown that sibling internalizing problems are related to more adolescent internalizing problems (Branje et al. 2004). Therefore, this study will also examine the link between sibling and adolescent depressive symptoms.

### Moderation by Sibling Relationship Quality

Identification or modeling processes may be particularly evident within a high-quality relationship, in which siblings feel strongly connected to each other and consequently identify themselves with each other (Brody 1998). Indeed, models tend to be more effective in the context of a warm and nurturing relationship (Whiteman et al. 2011). Within a negative relationship, adolescents may aspire to develop differently from their depressed sibling in a process of deidentification (Whiteman et al. 2007). Studies have indeed revealed that more conflict is linked to more differentiation (Whiteman and Christiansen 2008), which could result in less similarity in behavior. Thus, in addition to a direct effect of sibling depressive symptoms on adolescent depressive symptoms, perceived relationship quality may moderate this association.

### Sibling Status

Sibling modeling could also differ by sibling status (older/younger) because older and younger siblings may differently influence each other (Defoe et al. 2013). Because older siblings generally have a higher status in the family and more advanced skills, they are thought to be more influential models to their younger models than the other way around (Whiteman et al. 2011). Therefore, the impact of sibling depressive symptoms and the relationship with siblings may be stronger for younger siblings than for older siblings. However, previous research found no differences by sibling status in the relation between perceived sibling support and internalizing problems (Branje et al. 2004) or the association between conflict and depressive symptoms (Vogt Yuan 2009). Sibling status differences in the relations between power balance and adolescent depressive symptoms have not been examined. Therefore, in the current study, both the impact of older on younger sibling and the impact of younger on older siblings will be examined, allowing for exploration of potential differences based on sibling status.

### Comparing Influences of Sibling Relationship Quality and Sibling Depressive Symptoms

Although in previous research evidence was found for effects of quality of the sibling relationship as well as depressive symptoms of the sibling on adolescent depressive symptoms, most studies have examined these mechanisms separately, whereas there is much to be gained from studying them together. Only few studies have examined both mechanisms simultaneously. One exception concerns the study of Branje et al. (2004), which provided empirical support for linkages between perceived sibling support, sibling internalizing problems and adolescent internalizing problems. Their results suggest a slightly stronger effect of support on adolescents’ self-reported internalizing problems, compared to the effect of sibling internalizing problems. However, it should be noted that this study focused exclusively on perceived support, and not on conflict and power balance, whereas Buist et al. (2013)’s meta-analysis showed that conflict between siblings has the strongest effect on adolescent problem behavior. Based on these meta-analytic results, Buist et al (2013) concluded that it is important to also examine this particular characteristic of the sibling relationship. Therefore, the current study will add to existing knowledge by examining all three important sibling relationship indicators (support, conflict as well as power balance).

By examining the effect of sibling relationship quality as well as sibling depressive symptoms, their relative importance within the sibling context in predicting adolescent depressive symptoms can be compared. This knowledge can contribute to the fine-tuning of family-based interventions to prevent or reduce depressive symptoms among adolescents. If the results show that especially sibling depressive symptoms influence adolescent depressive symptoms, these should be the focus of interventions. Conversely, if the results demonstrate a stronger effect of sibling relationship quality, addressing support, conflict and power balance in the sibling relationship may be a better approach when attempting to decrease adolescent depressive symptoms.

### Longitudinal Patterns

Because both depressive symptoms and the sibling relationship are dynamic and change across adolescence (Updegraff et al. 2002) it is important to examine these processes longitudinally. As earlier studies have shown that siblings mutually influence each other’s emotional functioning (Gamble et al. 2011) it is essential to examine the direction of effects. For instance, adolescent depressive symptoms may not only be influenced by the quality of the sibling relationship, but these depressive symptoms may also evoke negative responses from siblings, resulting in a relative decrease in perceived relationship quality over time (Coyne et al. 1991). Without a longitudinal design, these mutual influences cannot be detected. A longitudinal design therefore expands current knowledge of over-time processes within the sibling relationship and their importance for emotional wellbeing during adolescence and vice versa. Therefore, the present study will examine longitudinal linkages between adolescent and sibling depressive symptoms and sibling relationship quality perceived by adolescents.

### Moderation by Dyadic Gender Composition

Dyadic gender composition may moderate the theorized longitudinal effects of sibling depressive symptoms and perceived sibling relationship quality on adolescent depressive symptoms. Same-gender dyads are generally closer and show greater similarity in problem behavior, such as delinquency, than mixed-gender dyads (Buist 2010). Limited evidence exists for similarity in depressive symptoms. In one of the few studies on this subject, the effects of perceived support from siblings and sibling depressive symptoms on adolescent depressive symptoms were not moderated by the dyadic gender composition (Branje et al. 2004). However, these results need replication, and it is unknown whether the effects of conflict and power balance on adolescent depressive symptoms are moderated by the dyadic gender composition. The current study will therefore explore whether the longitudinal effects of sibling depressive symptoms and perceived relationship quality on adolescent depressive symptoms differ by dyadic gender composition.

## Current Study

In the present longitudinal study, the first research aim was to examine the relative importance of sibling depressive symptoms and perceived sibling relationship quality in predicting adolescent depressive symptoms. Based on earlier work, no clear expectations were formulated concerning the relative importance of perceived sibling relationship quality and sibling depressive symptoms in predicting adolescent depressive symptoms. However, on the basis of attachment theory and earlier empirical findings, less perceived support, more conflict, and more power imbalance were expected to predict more adolescent depressive symptoms over time. More adolescent depressive symptoms were also expected to evoke negative responses from siblings, resulting in less perceived support and more perceived conflict. Due to lack of empirical evidence, no specific expectations were formulated whether adolescent depressive symptoms would predict changes in power balance in the sibling relationship. Furthermore, in line with the theory of sibling identification, higher levels of sibling depressive symptoms were expected to predict more adolescent depressive symptoms over time. The influence of older and younger siblings on each other’s depressive symptoms were expected to be similar in strength.

The second research aim was whether perceived sibling relationship quality moderated the effect of sibling depressive symptoms on adolescent depressive symptoms. Perceived relationship quality was expected to moderate the link between older and younger sibling depressive symptoms, in that the effect of sibling depressive symptoms on adolescent depressive symptoms was expected to be stronger when the perceived quality of the sibling relationship was higher (i.e., more support, less conflict, less power imbalance).

The third study aim was whether the effects of sibling relationship quality or sibling depressive symptoms on adolescent depressive symptoms differed by dyadic gender composition. Dyadic gender composition was not expected to moderate the effects of sibling support on adolescent depressive symptoms. Due to lack of theory and empirical studies, no clear expectations were formulated regarding this moderating effect for conflict or power balance.

## Methods

### Participants

This study used six waves of data from the ongoing longitudinal study Research on Adolescent Development And Relationships (RADAR; Van Lier et al. 2008) in which 497 Dutch adolescents between 13 and 18 years old and their siblings were followed across six measurement waves with one-year intervals. As the current study examined perceived longitudinal processes within the sibling dyad, only sibling dyads in which the same siblings participated across waves were included. This resulted in a final sample of 412 sibling dyads, in which younger siblings (56.6% boys) were on average 12.34 years old (*SD* = 1.22) and older siblings (46.6% boys) were on average 15.36 years old (*SD* = 2.28) at the first measurement. The average age gap between younger and older sibling was 3.03 years (*SD* = 1.80). Moreover, the sample consisted of 114 brother–brother dyads, 101 sister–sister dyads, 78 older brother–younger sister dyads and 119 older sister–younger brother dyads. Furthermore, 89.6% of the dyads lived in a family with a moderate to high socioeconomic status (SES), whereas 9.2% of the dyads lived within a family with a low SES.

Sibling dyads within this final sample did not differ from the 87 sibling dyads that were excluded because no or multiple siblings participated regarding adolescent gender (χ^2^(1, *N* = 497) = 0.01, *p* = 0.92, φ = −0.004) and adolescent age at the first measurement (*t*(495) = 1.26, *p* = 0.21, *d* = 0.11). Importantly, target adolescents who were included in the analyses did not differ significantly from those who were excluded with regard to the variables of interest, that is, dimensions of perceived relationship quality (*F*(18, 300) = 0.77, *p* = 0.74, η^2^ = 0.04) and depressive symptoms (*F*(6, 361) = 0.67, *p* = 0.68, η^2^ = 0.01). However, the final sample included significantly more children from a family with a medium to high SES (χ^2^(1, *N* = 497) = 5.67, *p* = 0.02, φ = −0.108) than those who were excluded, although this difference was small.

Attrition was relatively low in the present study. Of the 412 sibling dyads that participated at the first wave 405, 401, 396, 388 and 367 dyads participated at the consecutive five waves respectively. Thus, 10.9% of the 412 sibling dyads dropped out during the study. Per variable maximally 17.2% of the cases were missing in the sample. Little’s Missing Completely At Random (MCAR; Little 1988) test suggested that these missings were completely at random, χ^2^(1996) = 871.88, *p* > 0.99.

### Procedure

Through schools, target adolescents were approached and informed about the RADAR project. Siblings were invited to participate as well. Target adolescents, siblings and their parents gave written informed consent. As any sibling older than 10 years at Time 1 could participate, siblings were not necessarily closest in age. If more than one sibling was eligible to participate, families were free to choose which one. During annual home visits, trained interviewers provided questionnaires and gave verbal instructions. Both siblings filled out questionnaires at home simultaneously and received a monetary reward for each year they participated.

### Measures

For all measures, if less than 10%, missing item values were estimated by the Expectation Maximization (EM) procedure in SPSS.

#### Depressive symptoms

Both siblings reported on their own depressive symptoms on the Reynolds Adolescent Depression Scale—second edition (RADS-II; Reynolds 2000). They reported on the subscales Dysphoric mood (8 items, e.g. “I feel lonely”), Negative self-evaluation (8 items, e.g. “I feel I am bad”) and Somatic complaints (7 items, e.g. “I am tired”). Answers were given on a 4-point Likert scale (1 = *almost never* to 4 = *most of the time*). Some items were recoded to ensure that a higher score reflected more depressive symptoms. A total mean score of these subscales was used to measure the total amount of depressive symptoms. The RADS-II had a good internal consistency (alphas between 0.92 and 0.94 for both sibling reports across waves) and has good psychometric properties (Myers and Winters 2002; Osman et al. 2010).

#### Perceived sibling relationship quality

Both siblings reported on the perceived sibling relationship quality on the short version of the Network of Relationships Inventory questionnaire (NRI; Furman and Buhrmester 1985). The validity of the NRI has been demonstrated in previous research (Edens et al. 1999). Both adolescents reported on perceived support, conflict and power balance within the sibling dyad using the Support, Negative interaction and Power subscale. Answers were given on a 5-point Likert scale (1 = *a little or not at all* to 5 = *more is not possible*). Mean scores were used with higher scores reflecting more perceived support, more perceived conflict between siblings and more perceived power from sibling. The Support subscale consisted of 12 items, such as “How much does your sibling really care about you?” and had a good internal consistency (alphas between 0.84 and 0.87 across waves). The Negative interaction subscale consisted of six items, such as “How often do you and your sibling argue?” and had a good internal consistency (alphas between 0.94 and 0.95 across waves). The Power subscale consisted of six items, such as “How often does your sibling tell you what to do?” and had a good internal consistency (alphas between 0.88 and 0.93 across waves).

### Analytic Strategy

**Fig. 1:**
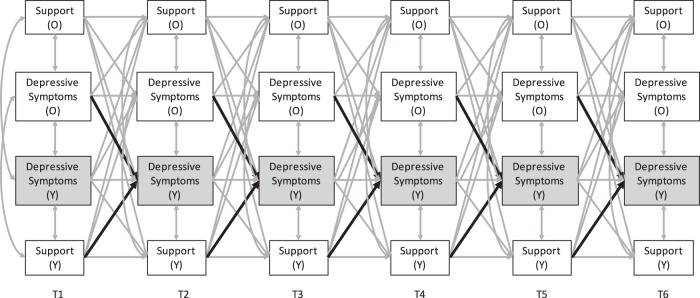
Main theorized cross-lagged panel model. *Note*. Older sibling (O) and younger sibling (Y) reports were used. Similar models were examined for perceived conflict and power

Before starting the analyses, the data set was restructured so that a distinction could be made between older and younger siblings. We first ran all models focusing on younger sibling outcomes, followed by models focusing on older sibling outcomes. To examine the hypothesized (bi)directional associations over time between adolescent depressive symptoms on the one hand and sibling depressive symptoms and perceived sibling relationship quality on the other hand, cross-lagged panel models were formulated (M*plus* version 8.1; Múthen and Múthen 1998–2017), as shown in Fig. 1. In all models, a Full Information Maximum Likelihood estimator was used that was robust to the non-normality of the data (Enders and Bandalos 2001; Múthen and Múthen 1998–2017).

Separate cross-lagged panel models were tested for each indicator of perceived quality of the sibling relationship (i.e. support, conflict and power balance). In each model, one-year stability effects, Time 1 correlations between the variables, correlated changes (i.e. correlated residuals within the other waves) and cross-lagged effects were estimated. Time 1 correlations indicate concurrent associations between older sibling depressive symptoms, younger sibling depressive symptoms and perceived relationship quality according to older and younger sibling. As Time 1 correlations and cross-lagged effects were controlled for, correlated residuals represent common changes in older and younger sibling depressive symptoms and perceived relationship quality. Controlling for relative stabilities of variables and concurrent associations, cross-lagged effects reflect longitudinal effects of, for instance, older sibling depressive symptoms on younger sibling depressive symptoms.

Hypothesized cross-lagged effects were estimated to examine effects of older sibling depressive symptoms and perceived relationship quality (according to older and younger sibling) on younger sibling depressive symptoms one year later (represented by the bold arrows in Fig. 1). To examine the relative importance of perceived sibling relationship quality on the one hand and older sibling depressive symptoms on the other hand in predicting younger sibling depressive symptoms, we standardized all variables and subsequently tested whether these cross-lagged effects (represented by the bold arrows in Fig. 1) could be constrained to be equal without significantly worsening model fit.

Moreover, the potential moderating effects of perceived relationship quality on the association between older sibling depressive symptoms on younger sibling depressive symptoms were examined. Hence, a centered interaction between relationship quality perceived by younger sibling and depressive symptoms of older sibling was estimated in predicting younger sibling depressive symptoms over time. We estimated and controlled for other longitudinal links (i.e. cross-lagged paths) between the constructs, and also for dyadic age differences between older and younger sibling by adding this variable as a covariate in the model because dyads differed in the age gap between siblings and previous studies have shown that dyads with small sibling age differences show stronger associations (Buist et al. 2013).

Multigroup analyses were conducted to examine moderation effects of dyadic gender composition by testing whether cross-lagged effects of older sibling depressive symptoms and perceived relationship quality on younger sibling depressive symptoms could be constrained to be equal for brother dyads, sister dyads and mixed-gender dyads without significantly worsening model fit.

To explore whether influence patterns were similar for younger versus older siblings, all models described above were also tested with older sibling depressive symptoms as outcome variables. So, in separate models, moderation effects of sibling relationship quality and dyadic gender composition were also tested for the prediction of *older* sibling depressive symptoms over time.

## Results

Mean levels and standard deviations of depressive symptoms, support, conflict and power imbalance as reported by both siblings are presented in Table 1. In general, both siblings reported relatively low levels of depressive symptoms (ranging from 1.51 to 1.71 on a 4-point scale). Furthermore, on average, siblings perceived relatively moderate levels of sibling support and low levels of conflict and power imbalance in their relationship. Over time younger sibling depressive symptoms first decreased and then increased moderately and older sibling depressive symptoms decreased more strongly. Perceived sibling support increased slightly over time, whereas perceived conflict between siblings and perceived power imbalance slightly decreased.

**Table 1:** Means and standard deviations of younger and older sibling depressive symptoms, perceived support, conflict and power imbalance

	T1	T2	T3	T4	T5	T6	
	*M (SD)*	*M (SD)*	*M (SD)*	*M (SD)*	*M (SD)*	*M (SD)*	*Over-time difference*
*Depressive symptoms*							
Younger sibling	1.61 (0.46)	1.51 (0.47)	1.51 (0.50)	1.54 (0.54)	1.54 (0.51)	1.57 (0.53)	*F*(5, 301) = 5.11, *p* < 0.001, η^2^ = 0.08
Older sibling	1.71 (0.49)	1.60 (0.50)	1.58 (0.50)	1.56 (0.50)	1.55 (0.48)	1.52 (0.48)	*F*(5, 295) = 9.01, *p* < 0.001, η^2^ = 0.13
*Sibling relationship quality*							
Support YO	3.19 (0.67)	3.26 (0.68)	3.26 (0.72)	3.30 (0.72)	3.33 (0.70)	2.20 (0.73)	*F*(5, 297) = 2.74, *p* = 0.02, η^2^ = 0.04
Conflict YO	2.28 (0.77)	2.23 (0.78)	2.19 (0.82)	2.07 (0.82)	1.97 (0.78)	1.89 (0.77)	*F*(5, 297) = 14.74, *p* < 0.001, η^2^ = 0.20
Power YO	2.38 (0.67)	2.34 (0.76)	2.29 (0.76)	2.28 (0.76)	2.18 (0.68)	2.15 (0.79)	*F*(5, 296) = 5.46, *p* < .001, η^2^ = 0.08
Support OY	3.11 (0.61)	3.16 (0.60)	3.22 (0.60)	3.26 (0.62)	3.27 (0.64)	3.34 (0.63)	*F*(5, 283) = 8.08, *p* < 0.001, η^2^ = 0.13
Conflict OY	2.38 (0.79)	2.29 (0.80)	2.23 (0.82)	2.13 (0.83)	1.92 (0.76)	1.81 (0.69)	*F*(5, 283) = 35,56, *p* < 0.001, η^2^ = 0.39
Power OY	1.73 (.58)	1.59 (0.50)	1.62 (.58)	1.59 (0.56)	1.61 (0.52)	1.64 (0.54)	*F*(5, 282) = 4.62, *p* < .001, η^2^ = 0.08

*Note*: *OY* older sibling report; *YO* younger sibling report

### Model Comparisons

With longitudinal models, it is important that variables are measurement-invariant. To test measurement invariance of the variables over time, alignment optimization was used (Asparouhov and Muthén 2014; Lomazzi 2018). These analyses showed that all factor loadings were invariant over six measurement waves and that only few small differences existed in factor intercepts, allowing for reliable testing of over-time associations between sibling relationship quality and adolescent depression^1^.

Next, separate models for perceived support, conflict, and power imbalance were constructed. For each model, a baseline model was formulated that included one-year stability paths, T1 correlations, correlated changes and cross-lagged effects. Moreover, all variables were regressed on the variable representing dyadic age differences between target adolescent and sibling in order to control for dyadic age differences by regressing. These baseline models for support (χ^2^(164) = 573.68, *p<* 0.001, *RMSEA* = 0.08, *CFI* = 0.91), conflict (χ^2^(164) = 527.99, *p<* 0.001, *RMSEA* = 0.07, *CFI* = 0.92) and power imbalance (χ^2^(164) = 581.84, *p<* 0.001, *RMSEA* = 0.08, *CFI* = 0.89) did not have a good fit. Adding two-year stability paths significantly improved the fit of the model for support (Δχ^2^(16) = 319.16, *p* < 0.001), the model for conflict (Δχ^2^(16) = 293.25, *p* < 0.001), and the model for power imbalance (Δχ^2^(16) = 371.34, *p* < 0.001). Furthermore, these improved models were time invariant as over-time stabilities and correlated changes (support Δχ_SB_^2^(52) = 67.51, *p* > 0.05; conflict (Δχ_SB_^2^(52) = 67.41, *p* > 0.05; power imbalance (Δχ_SB_^2^(52) = 47.95, *p* > 0.10) could be constrained to be equal over time without significantly worsening model fit. Cross-lagged effects were also largely time-invariant (support Δχ_SB_^2^(64) = 91.95, *p* < 0.05; conflict (Δχ_SB_^2^(64) = 74.85, *p* > 0.10; power imbalance (Δχ_SB_^2^(64) = 83.38, *p* > 0.05). To increase ease of interpretation, all these stabilities, correlated changes and cross-lagged effects were constrained to be equal across waves. The fit of these final models for support (χ^2^(264) = 412.99, *p<* 0.001, *RMSEA* = 0.04, *CFI* = 0.97), conflict (χ^2^(264) = 375.69, *p<* 0.001, *RMSEA* = 0.03, *CFI* = 0.98) and power imbalance (χ^2^(264) = 338.52, *p<* 0.001, *RMSEA* = 0.03, *CFI* = 0.98) was good.

### Associations Between Adolescent and Sibling Depressive Symptoms and Perceived Relationship Quality

The estimates of the final models for perceived support, conflict and power imbalance are displayed in Table 2. Due to the time invariance constraints, all stabilities, correlated residuals and cross-lagged paths were equal across time. Overall, effect sizes were modest. As displayed in this table, older and younger sibling depressive symptoms, and perceived support, conflict and power imbalance showed significant and moderate stability.

**Table 2:** Concurrent and longitudinal associations between older sibling (O) and younger sibling (Y) depressive symptoms and sibling relationship

	Support		Conflict		Power Imbalance	
	*B (SE)*	β	*B (SE)*	β	*B (SE)*	β
***Relative stability paths***						
Depressive symptoms (Y)	0.55 (0.03)	0.55^***^	0.55 (0.03)	0.55***	0.55 (0.03)	0.55***
Depressive symptoms (O)	0.56 (0.03)	0.56^***^	0.56 (0.03)	0.56***	0.56 (0.03)	0.56***
Sibling relationship (YO)	0.54 (0.03)	0.57^***^	0.48 (0.03)	0.51***	0.48 (0.02)	0.42***
Sibling relationship (OY)	0.56 (0.02)	0.57^***^	0.51 (0.03)	0.52***	0.41 (0.04)	0.46***
***T1 correlations***						
Depressive symptoms (Y) ↔						
Depressive symptoms (O)	0.02 (0.01)	0.10^*^	0.02 (0.01)	0.10*	0.02 (0.01)	0.10*
Sibling relationship (OY)	−0.02 (0.02)	−0.07	0.04 (0.02)	0.11*	0.01 (0.01)	0.02
Sibling relationship (YO)	−0.06 (0.02)	−0.19**	0.10 (0.02)	0.27***	0.03 (0.02)	0.10*
Depressive symptoms (O) ↔						
Sibling relationship (OY)	−0.06 (0.02)	−0.19***	0.09 (0.02)	0.24***	0.01 (0.02)	0.05
Sibling relationship (YO)	−0.00 (0.02)	−0.01	0.02 (0.02)	0.05	0.02 (0.02)	0.06
Sibling relationship (OY) ↔						
Sibling relationship (YO)	0.17 (0.03)	0.39***	0.29 (0.04)	0.47***	−0.01 (0.02)	−0.02
***Correlated residuals***						
Depressive symptoms (Y) ↔						
Depressive symptoms (O)	0.00 (0.00)	0.03	0.00 (0.00)	0.02	0.00 (0.00)	0.03
Sibling relationship (OY)	−0.01 (0.00)	−0.05*	0.01 (0.01)	0.04+	0.00 (0.00)	0.02
Sibling relationship (YO)	−0.02 (0.01)	−0.09***	0.03 (0.01)	0.12***	0.02 (0.01)	.08***
Depressive symptoms (O) ↔						
Sibling relationship (OY)	−0.02 (0.00)	−0.09***	0.24 (0.01)	0.10***	0.01 (0.00)	0.07**
Sibling relationship (YO)	−0.00 (0.00)	−0.00	0.01 (0.00)	0.04*	0.01 (0.01)	0.03+
Sibling relationship (OY) ↔						
Sibling relationship (YO)	0.04 (0.01)	0.16***	0.09 (0.01)	0.24***	0.01 (0.01)	0.03
***Cross-lagged paths***						
Depressive symptoms (Y)						
Depressive symptoms (O)	0.06 (0.02)	0.06^**^	0.06 (0.02)	0.07**	0.06 (0.02)	0.06**
Sibling relationship (YO)	−0.01 (0.01)	−0.01	0.00 (0.01)	0.01	0.01 (0.01)	0.01
Sibling relationship (OY)	−0.00 (0.01)	−0.00	−0.01 (0.01)	−0.01	0.01 (0.02)	0.01
Depressive symptoms (O)						
Depressive symptoms (Y)	0.04 (0.02)	.04^**^	0.04 (0.02)	0.04**	0.05 (0.02)	0.04**
Sibling relationship (OY)	0.01 (0.01)	0.01	0.01 (0.01)	0.01	0.01 (0.02)	0.01
Sibling relationship (YO)	−0.01 (0.01)	−0.01	0.00 (0.01)	0.00	−0.01 (0.01)	−0.02
Sibling relationship (YO)						
Depressive symptoms (Y)	−0.06 (0.02)	−0.04*	0.04 (0.03)	0.02	0.03 (0.03)	0.02
Depressive symptoms (O)	−0.02 (0.02)	−0.02	0.03 (0.03)	0.02	0.03 (0.03)	0.02
Sibling relationship (OY)	0.08 (0.02)	0.08***	0.08 (0.02)	0.08**	−0.01 (0.03)	−0.01
Sibling relationship (OY)						
Depressive symptoms (O)	−0.03 (0.02)	−0.02	0.05 (0.03)	0.03	0.02 (0.02)	0.02
Depressive symptoms (Y)	0.08 (0.02)	0.06***	−0.07 (0.03)	−0.04*	0.02 (0.02)	0.02
Sibling relationship (YO)	0.09 (0.02)	0.10***	0.09 (0.02)	0.09***	−0.01 (0.01)	−0.02

*Note*: Due to the time invariance constraints, all stabilities, correlated residuals and cross-lagged paths are equal across time

*p < 0.05; **p < 0.01; ***p < 0.001

As expected, higher levels of younger sibling depressive symptoms were significantly related to less perceived support and more perceived conflict and power imbalance. Results for older sibling depressive symptoms were similar, with one exception: Their depressive symptoms were also significantly related to less support and more conflict, but the correlation with power imbalance was not significant.

Consistent with expectations, within the first wave, higher levels of younger sibling depressive symptoms were significantly but weakly related to more older sibling depressive symptoms. Older and younger sibling’s perceptions of sibling relationship quality were significantly intercorrelated for support and conflict, but not for power imbalance.

### Longitudinal Linkages Between Adolescent and Sibling Depressive Symptoms and Perceived Relationship Quality

As indicated by the correlated residuals, changes in younger and older sibling depressive symptoms were significantly related to changes in perceived support, conflict, and power imbalance. However, changes in younger sibling depressive symptoms were not linked to changes in older sibling depressive symptoms. Additionally, changes in both adolescents’ perception of support and conflict were interrelated, but not for power imbalance.

Cross-lagged effects showed that, in line with expectations, younger sibling depressive symptoms were significantly predicted by older sibling depressive symptoms one year earlier. In contrast to expectations, younger sibling depressive symptoms over time were not significantly predicted by perceived support, conflict and power imbalance. Identical results were found for the prediction of older sibling depressive symptoms, which were significantly predicted by younger sibling depressive symptoms, but not by perceived support, conflict and power imbalance.

Furthermore, the effects of depressive symptoms on perceived sibling relationship quality over time were examined. For younger siblings, more younger sibling depressive symptoms significantly predicted less perceived support one year later. No significant effect for conflict or power imbalance was found. For older siblings, their own depressive symptoms did not predict perceived sibling relationship quality over time. However, support and conflict as perceived by the older sibling were significantly predicted by younger sibling depressive symptoms: More younger sibling depressive symptoms predicted more support and less conflict as perceived by the older sibling one year later. No significant effects of younger nor older sibling depressive symptoms on perceived power imbalance were found.

Lastly, support and conflict as perceived by older siblings significantly affected support and conflict as perceived by younger siblings one year later, and vice versa. Again, no significant effects concerning sibling power imbalance were found.

### Comparing Influences of Sibling Relationship Quality and Sibling Depressive Symptoms

Subsequent models tested whether sibling depressive symptoms on the one hand and relationship quality on the other hand could be constrained to be equal in predicting depressive symptoms over time. To allow for meaningful comparison, standardization of scores was applied in these models. The results showed that for the prediction of younger sibling depressive symptoms, constraining the effects of older sibling depressive symptoms and sibling relationship quality as perceived by the younger sibling led to a significantly better-fitting model for support (Δχ^2^ (1) = −7.86, *p* < 0.001), similar-fitting model for conflict (Δχ^2^ (1) = 2.30, *p* > 0.05) and significantly worse-fitting model for power imbalance (Δχ^2^ (1) = 16.19, *p* < 0.001).

For the prediction of older sibling depressive symptoms, constraining the effects of younger sibling depressive symptoms and sibling relationship quality as perceived by the older sibling led to a significantly better-fitting model for support (Δχ^2^ (1) = −10.01, *p* < 0.001), similar-fitting model for conflict (Δχ^2^ (1) = −1.86, *p* > 0.05) and significantly worse-fitting model for power imbalance (Δχ^2^ (1) = 14.73, *p* < 0.001).

So, in the prediction of adolescent depressive symptoms, the over-time effects of sibling depressive symptoms are significantly stronger than the over-time effects of power imbalance, but similar to the over-time effects of sibling support or sibling conflict.

### Moderation by Sibling Relationship Quality

Next, the potential *moderating effect of perceived relationship quality* on the effect of older sibling depressive symptoms on younger sibling depressive symptoms was tested. However, contrary to expectations, none of the interaction terms were significant: perceived support, conflict and power imbalance did not moderate the effect of older sibling depressive symptoms on younger sibling depressive symptoms.

These moderation models were also tested with older sibling depressive symptoms as outcome variables. The results of these analyses were identical to those with younger sibling depressive symptoms as outcome variables: Perceived support, conflict, and power imbalance did not moderate the effect of younger sibling depressive symptoms on older sibling depressive symptoms.

### Moderation by Dyadic Gender Composition

Next, the possible *moderating effect of dyadic gender composition* on the longitudinal effects of sibling depressive symptoms and perceived sibling relationship quality on adolescent depressive symptoms differed by dyadic gender composition. Multigroup analyses were conducted, testing whether these cross-lagged effects could be constrained to be equal for dyadic gender composition without significantly worsening model fit. These models were tested separately for younger and older sibling depressive symptoms as outcome variables. The results show that dyadic gender composition did not moderate cross-lagged effects in any of these models. This implies that the effects of sibling depressive symptoms and perceived relationship quality on adolescent depressive symptoms did not differ by adolescent dyadic gender composition.

### Sensitivity Analysis

The robustness of these findings was checked by examining an alternative model in which the potential moderating effects were examined between sibling relationship quality according to younger sibling and according to older sibling in predicting adolescent depressive symptoms. Similar to the models concerning the moderating effects between sibling relationship quality and sibling depressive symptoms on adolescent depressive behavior, a centered interaction between relationship quality perceived by younger sibling and by older sibling was estimated in predicting adolescent depressive symptoms over time. Again, cross-lagged paths and dyadic age differences between older and younger sibling were controlled for. These models were formulated separately for younger and older sibling depressive symptoms as outcomes, and separately for perceived support, conflict and power imbalance (so six models in total).

None of the interaction terms between older and younger sibling perceived relationship quality significantly predicted adolescent depressive symptoms (for younger or older sibling). The conclusion is therefore that there are no moderating effects between perceived relationship quality of older and younger siblings in predicting adolescent depressive symptoms.

## Discussion

The sibling relationship has an important impact on children’s emotional functioning (Buist et al. 2013). During adolescence, the sibling relationship changes (McHale et al. 2006) and depressive symptoms increase (Costello et al. 2011). However, whether and how sibling relationship quality affects adolescent depressive symptoms over time is not yet clear. Whereas support, conflict and power have been identified as salient dimensions of the sibling relationship (Campione-Barr 2017), research on how these three dimensions (particularly power balance) are linked to adolescent depressive symptoms over time is scarce or missing. Additionally, the possibility that adolescent depressive symptoms may also be affected by depressive symptoms of their sibling has not been examined longitudinally throughout adolescence. Using a six-wave longitudinal design with a large community sample of 412 sibling pairs, this study addresses these gaps in knowledge by providing insight into the relative importance of perceived sibling relationship quality and sibling depressive symptoms in predicting adolescent depressive symptoms. Because sibling influence may differ as a function of perceived sibling relationship quality (Brody 1998) and dyadic gender composition (Buist 2010), this study also examined these potential moderating effects. Overall, the present study found that adolescent depressive symptoms were more strongly influenced by sibling depressive symptoms than by sibling relationship quality, and that these influences did not differ as a function of sibling relationship quality and dyadic gender combination, as discussed in more detail below.

### Relative Importance of Sibling Relationship Quality and Sibling Depressive Symptoms

The results seem to indicate that the perceived quality of the sibling relationship is less important in predicting adolescent depressive symptoms over time when sibling depressive symptoms are taken into account, although one has to keep in mind that the difference in effect size between perceived sibling relationship quality and sibling depressive symptoms was only significant in the case of power imbalance. Unexpectedly, although perceived sibling relationship quality and depressive symptoms were related concurrently, perceived sibling relationship quality did not predict younger depressive symptoms one year later. These findings are not completely in accordance with previous research and attachment theory (Bowlby 1969), which suggest that children whose interpersonal relationships are low in quality will show negative developmental outcomes.

However, the results do show concurrent associations, which suggest ongoing short-term dynamics through which relationships and depressive symptoms affect each other. It is possible that perceived sibling relationship quality is related to adolescent depressive symptoms on the short term but does not predict these depressive symptoms one year later. To further examine these short-term versus long-term patterns, it would be insightful to conduct a similar study with smaller time intervals.

Furthermore, more younger sibling depressive symptoms were related to less perceived support by the younger sibling but to more support and less conflict as perceived by the older sibling one year later. The fact that depressed younger siblings perceive less support from their older sibling may reflect a perception bias: They may experience support more negatively. The results concerning perception of the older sibling concerning increased support and decreased conflict are not in line with interpersonal theory on depression positing that depressed individuals eventually elicit negative responses from their environment (Coyne et al. 1991), possibly resulting in a decrease of perceived relationship quality over time. It may well be that this hypothesized mechanism applies to more distant relationships (e.g. peers, teachers) rather than the close sibling relationship. In the sibling relationship, older siblings seem to respond to depressive symptoms of their younger sibling by trying to be more supportive and less negative in their behavior towards their younger sibling. It is doubtful whether younger siblings actually feel supported, given the fact that they perceive *lower* support from their siblings one year later.

In line with sibling identification theory (Bank and Kahn 1982), the results show that adolescents whose sibling stated more depressive symptoms reported more depressive symptoms one year later. These longitudinal findings seem to be in line with findings of a previous study (Branje et al. 2004), which showed that whereas both sibling support and sibling internalizing problems were concurrently related to adolescent internalizing problems, only sibling internalizing problems had an effect over time. It is possible that depressive feelings of siblings are present and distinct to such an extent that they influence the mood (i.e., depressive symptoms) of the adolescent, above and beyond the effects of the perceived characteristics of the sibling relationship. Thus, depressed feelings might be contagious in a sense. Another explanation for these findings could be co-rumination (Stone et al. 2011). Siblings may talk to each other about their negative feelings and problems to such an extent that it may increase the risk for depressive symptoms. However, co-rumination was not measured in the current study. Future research should examine the precise relational processes (including co-rumination) through which siblings influence each other’s depressive symptoms during adolescence to obtain more understanding and draw firmer conclusions.

Concerning potential differences between older and younger sibling influence patterns, consistent with expectations, only very few differences were found. The results showed that overall, results were almost identical for older versus younger sibling depressive symptoms as outcome measure. This implicates bidirectional influences between older and younger siblings, possibly reflecting the more egalitarian sibling relationships during and after adolescence. Another intriguing possibility is that sibling influence is not mainly driven by sibling status or birth order, but by degree of depressive symptoms. So, it could be that the sibling with the highest level of depressive symptoms is driving the influence, which is in some cases the older and in other cases the younger sibling. This was not examined in the present study, but for future studies it could be insightful to examine this possible pattern.

### Moderation by Sibling Relationship Quality

Contrary to expectations, this longitudinal effect of sibling depressive symptoms on adolescent depressive symptoms was not moderated by perceived sibling relationship quality. This implies that regardless of the perceived quality of the sibling relationship, sibling depressive symptoms predicted more adolescent depressive symptoms one year later. Thus, no support was found for the hypothesized sibling identification mechanism in which this effect would be stronger in high quality sibling relationships due to resulting feelings of connectedness and identification. Yet, these conclusions should be drawn with caution, as similarities between siblings in depressive symptoms were examined instead of actual identification processes. Future research should examine the extent to which siblings identify themselves with each other to investigate these sibling identification processes and their relevance for depressive symptoms among adolescents (Whiteman et al. 2007). It is also possible that identification processes are strengthened by perceived similarity between siblings, for example concerning personality. It would be insightful to examine whether adolescents are more strongly influenced by (relationships with) siblings that are more similar to themselves in personality.

### Moderation by Dyadic Gender Composition

In line with previous research, longitudinal effects of sibling depressive symptoms and perceived sibling relationship quality on adolescent depressive symptoms were not moderated by dyadic gender composition (Branje et al. 2004). This implies that the effects were equivalent for different dyadic gender compositions. Hence, the findings seem to reflect universal patterns of association among adolescents, regardless of gender composition of the sibling dyad.

### Strengths and Limitations

The present study had several limitations. The first limitation concerns the use of self-reports to examine depressive symptoms, support, conflict and power imbalance. How adolescents perceive the quality of the sibling relationship may depend on their emotional wellbeing (Branje et al. 2002). Adolescents with depressive symptoms may perceive their sibling’s behaviors as less positive, for instance because they experience less support. However, this perception might be particularly important because it also influences adolescent emotional wellbeing. Moreover, there are indications that adolescents are the best informants of their own depressive symptoms as these processes are often internal and may go largely unnoticed by others. For example, research has demonstrated that parents underestimate problem behavior of their children, especially internalizing problems (Verhulst and Koot 1995). Some studies have also indicated that child perceptions of family relationships may be stronger and more reliable predictors of child adjustment than parental perceptions (Glasgow et al. 1997). Additionally, both siblings’ perspectives concerning their depressive symptoms and of the sibling relationship were included, instead of relying on just one perspective.

Sibling identification and modeling processes were introduced as an explanation for the links between older and younger sibling depressive symptoms. However, modeling nor identification with siblings were not directly measured. Future research should take this into account, providing more insight into the exact processes of sibling influence in the context of depressive symptoms.

Another limitation concerns the focus on solely the sibling relationship. As we did not control for parental and peer influences, from this study it remains unknown whether siblings have a unique effect on adolescent depressive symptoms above and beyond potential effects of others, such as parents and friends. Whereas similar studies demonstrated an effect of the sibling context above and beyond that of parents and friends concerning externalizing behavior (Defoe et al. 2013) and beyond that of parents concerning internalizing and externalizing behavior (Buist et al. 2011), future research should examine these processes in the development of depressive symptoms during adolescence by controlling for other influences to examine the relative importance of siblings. Furthermore, variables that were not assessed in the present study might influence depressive symptoms and perceived sibling relationship quality (Kim et al. 2007). For instance, parental depressive symptoms or parental divorce may relate to more adolescent and sibling depressive symptoms and also affect perceived sibling relationship quality. Moreover, genetic resemblance between adolescents and siblings could be an explanation for the associations between siblings in self-reported depressive symptoms. Therefore, studies should include genetically similar and dissimilar siblings to draw firm conclusions.

It is important to note that the adolescents in the current study reported relatively low levels of depressive symptoms. Therefore, the results cannot be generalized to clinically depressed adolescents. However, as the current study showed associations in a sample with relatively low levels of depressive symptoms, associations could possibly be even stronger within a sample of clinically depressed adolescents. Future research should examine whether similar associations exist among clinically depressed adolescents.

Last, the one-year interval between measurements may be too long to understand the precise associations within the sibling relationship, especially regarding relationship quality. As adolescence is characterized by changes, smaller intervals might be needed to capture these changes. A daily diary approach, in which participants report daily on their emotions and relationship, could provide an interesting approach for future research.

The present study also has a number of important strengths. First of all, the study used a six-wave longitudinal design, which allowed for an examination of the direction of effects between older and younger sibling depressive symptoms and perceived sibling relationship quality. Second, this study was one of the first to longitudinally examine the relative importance of perceived sibling relationship quality on the one hand and sibling depressive symptoms on the other hand in predicting adolescent depressive symptoms. Other strengths concern the relatively large sample size, the inclusion of reports of both siblings concerning their relationship, and the focus on three important dimensions of the sibling relationship, which enabled the examination of specific characteristics of the sibling relationship.

## Conclusion

Earlier work has examined depression in the sibling context, but knowledge about the over-time processes between sibling relationship quality, sibling depression and adolescent depression is lacking. This study examined the relative importance of perceived sibling relationship quality on the one hand and sibling depressive symptoms on the other hand in predicting adolescent depressive symptoms over time. Additionally, the potential moderating effects of sibling relationship quality and dyadic gender composition were examined to provide new insights into the universality of these influences. The study showed that only sibling depressive symptoms (and not sibling relationship quality) predicted adolescent depressive symptoms over time, regardless of sibling relationship quality and dyadic gender composition. The implications of this study are that especially depressive symptoms of siblings should be considered a risk factor for depressive symptoms during adolescence. Considering the fact that depressive symptoms tend to increase during adolescence (Costello et al. 2011) and the fact that adolescent depressive symptoms may be predictive of depression in adulthood (Birmaher et al. 2004), these findings may help fine-tune interventions to prevent or reduce depressive symptoms during adolescence and therefore improve quality of life for adolescents and adults.

## Footnotes

1 Syntax and output of these analyses are available from first author.
